# A Phase 2 randomised study to establish efficacy, safety and dosing of a novel oral cathepsin C inhibitor, BI 1291583, in adults with bronchiectasis: Airleaf

**DOI:** 10.1183/23120541.00633-2022

**Published:** 2023-06-26

**Authors:** James D. Chalmers, Abhya Gupta, Sanjay H. Chotirmall, April Armstrong, Peter Eickholz, Naoki Hasegawa, Pamela J. McShane, Anne E. O'Donnell, Michal Shteinberg, Henrik Watz, Anastasia Eleftheraki, Claudia Diefenbach, Wiebke Sauter

**Affiliations:** 1 Division of Molecular and Clinical Medicine, University of Dundee, Dundee, UK; 2 Boehringer Ingelheim International GmbH, Biberach, Germany; 3 Lee Kong Chian School of Medicine, Nanyang Technological University, Singapore, Singapore; 4 Department of Respiratory and Critical Care Medicine, Tan Tock Seng Hospital, Singapore, Singapore; 5 University of Southern California, Los Angeles, CA, USA; 6 Department of Periodontology, Goethe University Frankfurt, Frankfurt, Germany; 7 Department of Infectious Diseases, Keio University, Tokyo, Japan; 8 University of Texas Health Science Center at Tyler, Tyler, TX, USA; 9 Georgetown University Medical Center, Washington, DC, USA; 10 Lady Davis Carmel Medical Center, Haifa, Israel; 11 Pulmonary Research Institute at LungenClinic Grosshansdorf, Airway Research Center North (ARCN), German Center for Lung Research (DZL), Grosshansdorf, Germany

## Abstract

New therapies are needed to prevent exacerbations, improve quality of life and slow disease progression in bronchiectasis. Inhibition of cathepsin C (CatC) activity has the potential to decrease activation of neutrophil-derived serine proteases in patients with bronchiectasis, thereby reducing airway inflammation, improving symptoms, reducing exacerbations and preventing further airway damage. Here we present the design of a phase 2 trial (Airleaf™; NCT05238675) assessing the efficacy and safety of a novel CatC inhibitor, BI 1291583, in adult patients with bronchiectasis. This multinational, randomised, double-blind, placebo-controlled, parallel-group, dose-finding study has a screening period of at least 6 weeks, a treatment period of 24–48 weeks and a follow-up period of 4 weeks. ∼240 adults with bronchiectasis of multiple aetiologies will be randomised to placebo once daily, or BI 1291583 1 mg once daily, 2.5 mg once daily or 5 mg once daily in a 2:1:1:2 ratio, stratified by *Pseudomonas aeruginosa* infection and maintenance use of macrolides. The primary efficacy objective is to evaluate the dose–response relationship for the three oral doses of BI 1291583 *versus* placebo on time to first pulmonary exacerbation up to Week 48 (the primary end-point). Efficacy will be assessed using exacerbations, patient-reported outcomes, measures of symptoms, sputum neutrophil elastase activity and pulmonary function testing. Safety assessment will include adverse event reporting, physical examination, monitoring of vital signs, safety laboratory parameters, 12-lead electrocardiogram, and periodontal and dermatological assessments. If efficacy and safety are demonstrated, results will support further investigation of BI 1291583 in phase 3 trials.

## Abstract 2

**Airleaf is a phase 2 study investigating the efficacy, safety and dosing of BI 1291583, a novel cathepsin C inhibitor, in bronchiectasis. The study is actively recruiting and results will support further investigation in phase 3 trials.** https://bit.ly/3G8fTzo

## Introduction

Bronchiectasis is a heterogeneous respiratory syndrome characterised by chronic airway inflammation; abnormal, scarred and irreversibly dilated bronchi; mucus plugging and subsequent airflow obstruction, facilitating bacterial infection [1–4]. The pathogenesis of bronchiectasis is not fully understood; however, once established, patients show evidence of chronic inflammation, infection, impaired mucociliary clearance and progressive structural lung damage. The complex interaction between these features (the so-called “vicious vortex”) leads to exacerbations and decline in pulmonary function, with associated morbidity and mortality [5].

Bronchiectasis has been described as an emerging global epidemic [6], with prevalence and incidence rates increasing worldwide [7, 8]. Prevalence increases with age and female sex [8, 9], and there is geographical heterogeneity in underlying disease aetiology [10], which ranges from well-characterised genetic diseases such as cystic fibrosis and primary ciliary dyskinesia, to a range of autoimmune diseases (for example, inflammatory bowel disease or rheumatoid arthritis), hypersensitivity disorders such as allergic bronchopulmonary aspergillosis, immunodeficiencies, and chronic airway inflammatory diseases such as COPD and asthma [5]. Each underlying aetiology considered individually may be rare but taken together bronchiectasis occurs in up to 566 people per 100  000 (0.6%) [11], with the UK reporting the highest global prevalence and incidence (566.1 per 100 000 women and 485.5 per 100 000 men, and 35.2 per 100 000 person-years in women and 26.9 per 100 000 person-years in men, respectively [7]). In addition to significant morbidity and mortality, bronchiectasis imposes a significant disease burden on patients due to reduced quality of life [12]. The disease also has a significant economic impact, including increased healthcare utilisation and costs, and loss of productivity [13].

**FIGURE 1:**
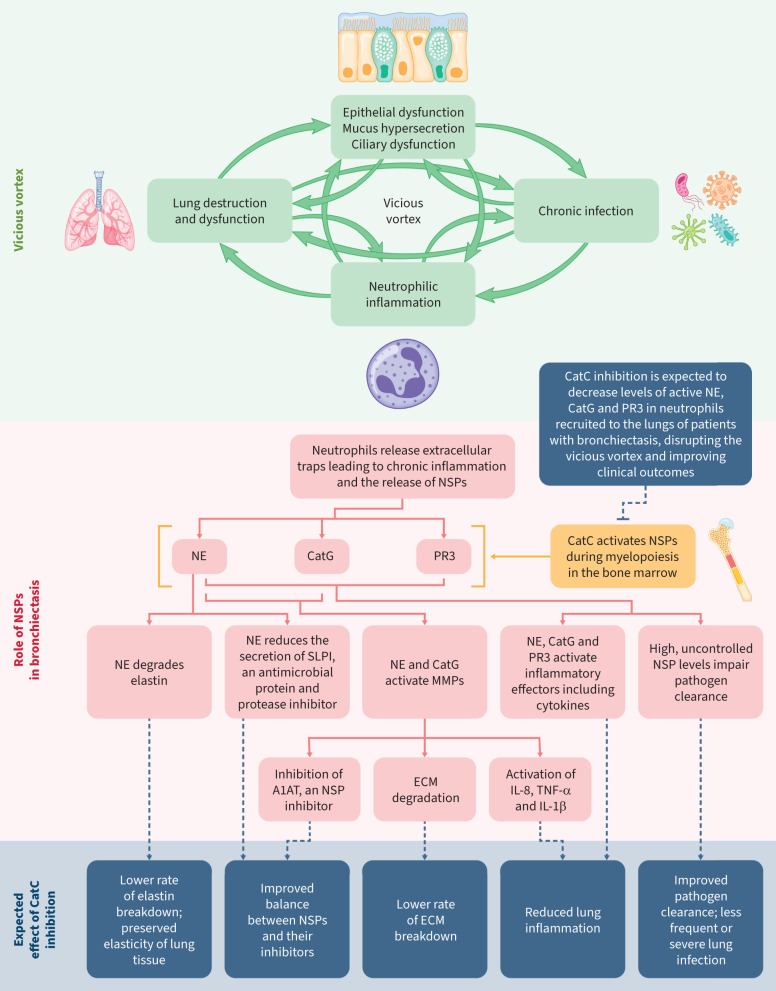
The “vicious vortex” in bronchiectasis, the key role of neutrophilic inflammation and the expected effects of inhibiting cathepsin C during myelopoiesis. A1AT: α-1 antitrypsin; CatC: cathepsin C; CatG: cathepsin G; ECM: extracellular matrix; IL-1β: interleukin-1β; IL-8: interleukin-8; MMP: matrix metallopeptidase; NE: neutrophil elastase; NSP: neutrophil-derived serine protease; PR3: proteinase 3; SLPI: secretory leukocyte peptidase inhibitor; TNF-α: tumour necrosis factor-α.

Neutrophils are abundant in the airways of patients with bronchiectasis, neutrophilic inflammation is a central feature of the disease (figure 1), and the extent of this inflammation is associated with disease severity and progression [14, 15]. While neutrophilic inflammation is closely linked to infection, inflammation can also progress in the absence of infection. In many cases of the disease, antibiotic treatment is insufficient to control infection [16].

Release of inflammatory effectors from neutrophils contributes to the inflammatory environment [17]. An imbalance between neutrophil-derived serine proteases (NSPs) (neutrophil elastase (NE), proteinase 3 (PR3) and cathepsin G (CatG)) and their inhibitors has been implicated in many chronic inflammatory respiratory diseases [18–21], including bronchiectasis [7]. Sputum NE activity was shown to be associated with increased risk and frequency of exacerbations, infections, hospitalisations and mortality [22, 23]. Levels of PR3 were found to be raised in patients with bronchiectasis during exacerbations compared with stable disease, correlating with levels of NE [24]. CatG activity was also found to cause dysfunction of ciliated cells and destruction of airway epithelium in patients with bronchiectasis, and activity correlated with disease severity [25].

NE, PR3 and CatG are activated by cathepsin C (CatC) (also known as dipeptidyl peptidase 1) during myelopoiesis in the bone marrow [26]. Inhibition of CatC is therefore expected to result in decreased levels of active NE, CatG and PR3 in neutrophils recruited to the lungs of patients with bronchiectasis. Since high, uncontrolled NSP levels have been shown to impair defence against bacterial infection, impair mucociliary clearance, promote mucus hypersecretion, and degrade elastin and other extracellular matrix components [7], effective blockade of NSPs could ameliorate each component of the vicious vortex. Interrupting the vicious vortex, possibly at multiple locations, through CatC inhibition, will likely reduce inflammation, lung destruction, mucus production and infection, and have secondary anti-inflammatory effects (figure 1).

No drug is licensed for the treatment of bronchiectasis. Therefore, there is a high unmet need for a novel bronchiectasis treatment that reduces aberrant inflammation, addresses exacerbations, and improves symptoms and patient quality of life.

BI 1291583 is a novel CatC inhibitor under investigation as a potential disease-modifying therapy for patients with bronchiectasis. *In vitro* and *in vivo* preclinical analyses demonstrated that BI 1291583 is a reversible, highly potent and highly selective inhibitor of CatC, with downstream dose-dependent effects on the production of active NE and PR3 after lipopolysaccharide challenge, and a high bone marrow-to-plasma distribution ratio. Five phase 1 trials in healthy volunteers, with dosing regimens of BI 1291583 based on effective dose calculations from the above preclinical analyses, demonstrated a good safety and tolerability profile up to 40 mg single doses and up to 10 mg multiple doses [27]. Taking the drug with food did not affect systemic exposure, and there was a low (up to twofold) increase in exposure with coadministration with itraconazole (a strong cytochrome and P-glycoprotein inhibitor) [27]. Adverse events of special interest (AESIs) related to skin, reported in previous trials of other CatC inhibitors in bronchiectasis [28–30], were observed more frequently in treated participants overall; however, cases were mild and those related to study drug were low (single cases except in the 5 mg dose), and were reported in both placebo and treated groups with the same frequency [27].

Based on the promising preclinical and phase 1 results, a multinational, randomised, double-blind, placebo-controlled, parallel-group, dose-finding phase 2 study in adult patients with bronchiectasis is now underway. Here we present and discuss the trial methodology.

## Research methods

### Study design

**FIGURE 2:**
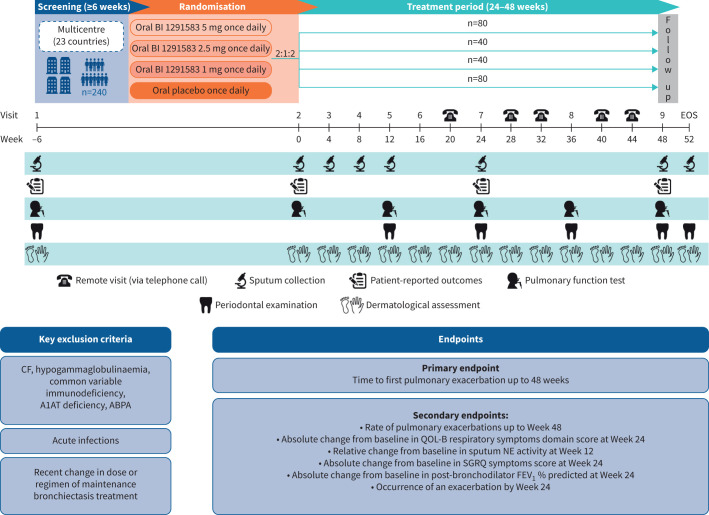
Study design. A1AT: α-1 antitrypsin; ABPA: allergic bronchopulmonary aspergillosis; CF: cystic fibrosis; EOS: end of study; FEV_1_: forced expiratory volume in 1 s; NE: neutrophil elastase; QOL-B: Quality of Life Questionnaire-Bronchiectasis; SGRQ: St George's Respiratory Questionnaire.

This phase 2 study (Airleaf™; NCT05238675) is a multinational, randomised, double-blind, placebo-controlled, parallel-group, dose-finding study that will be conducted in ∼23 countries. The study will comprise a screening period of at least 6 weeks, a treatment period of at least 24 weeks and up to 48 weeks, and a follow-up period of 4 weeks (figure 2).

Approximately 240 adults with bronchiectasis will be randomised in a 2:1:1:2 ratio, respectively, to placebo once daily (n=80), BI 1291583 1 mg once daily (n=40), BI 1291583 2.5 mg once daily (n=40) or BI 1291583 5 mg once daily (n=80). The randomisation will be stratified by *Pseudomonas aeruginosa* infection (yes/no) and by macrolide antibiotic maintenance (yes/no). Maintenance therapy with oral or inhaled antibiotics will be allowed if the patient is on stable treatment >3 months prior to randomisation and if an exacerbation occurred in the past year while being on that therapy. Maintenance therapy with bronchodilators will also be permitted during the treatment period, except 6–24 h prior to pulmonary function tests. A list of the baseline clinical data to be collected can be found in the supplementary material.

Once randomised, patients will be treated for at least 24 weeks and up to 48 weeks. At the planned end of treatment date of the last patient randomised (24 weeks), end of treatment will apply to all ongoing patients, and they will conclude study participation (end of study). Patients who prematurely and permanently discontinue study medication will be asked to attend future visits as scheduled.

### Objectives and end-points

The primary efficacy objective is to evaluate the dose–response relationship for oral 1 mg, 2.5 mg or 5 mg once daily BI 1291583 *versus* placebo on time to first pulmonary exacerbation up to Week 48 after first drug administration (primary end-point). Secondary objectives are to test the superiority of BI 1291583 5 mg *versus* placebo on the primary end-point and on the key secondary end-point (rate of pulmonary exacerbations up to Week 48), performed in a hierarchical order. Additional secondary efficacy end-points, along with safety, further clinical and pharmacokinetic end-points are described in table 1.

**TABLE 1:** Additional secondary efficacy, and safety, further clinical and pharmacokinetic end-points

**Secondary efficacy**	**Safety**	**Further clinical** **^#^**	**Pharmacokinetic**
Absolute change from baseline in QOL-B domain scores at Week 24 after first drug administration	Percentage of patients with treatment-emergent AEs up to the end of the REP (Week 52)	Change from baseline in cough frequency	C_pre,N_ C_pre,ss_
Relative change from baseline in NE activity in sputum at Week 12 after first drug administration	Physical examination, vital signs, safety laboratory parameters, 12-lead ECG	Change from baseline in PRO total and domain scores, and VAS scores for cough severity, cough urgency and shortness of breath	
Absolute change from baseline in SGRQ symptoms score at Week 24 after first drug administration		Number of subjects hospitalised due to pulmonary exacerbations	
Absolute change from baseline in FEV_1_ % predicted at Week 24 after first drug administration	Periodontal and dermatological assessments		
Occurrence of an exacerbation by Week 24 after first drug administration			

QOL-B: Quality of Life Questionnaire-Bronchiectasis; AE: adverse event; REP: residual effects period; C_pre,N_: pre-dose BI 1291583 plasma concentration immediately before administration of the Nth dose after N−1 doses were administered; C_pre,ss_: pre-dose BI 1291583 plasma concentration immediately before administration at pharmacokinetic steady state; NE: neutrophil elastase; ECG: electrocardiogram; PRO: patient-reported outcome; VAS: visual analogue scale; SGRQ: St. George's Respiratory Questionnaire; FEV_1_: forced expiratory volume in 1 s. **^#^**: at Week 24.

Additional objectives of the trial are to confirm BI 1291583-mediated reduction of NE activity in sputum at Week 12, assess lung function and quality of life after 24 weeks of treatment, assess additional exploratory measures of efficacy (including exacerbations, lung function and patient-reported outcomes), and evaluate pharmacokinetics, changes over time in blood and sputum biomarkers, safety and tolerability.

### Outcome assessments

Efficacy will be assessed using measures of disease worsening (time to first, and rate of, pulmonary exacerbation), patient-reported outcomes (*e.g.* Quality of Life Bronchiectasis Questionnaire, St. George's Respiratory Questionnaire (SGRQ) symptoms score and visual analogue scales), relative change from baseline in sputum NE activity and pulmonary function (forced expiratory volume in 1 s (FEV_1_) % predicted).

Safety will be monitored throughout the study by assessment of adverse events, physical examination, monitoring of vital signs, safety laboratory parameters and 12-lead electrocardiogram. As in the phase 1 trials of BI 1291583 [27], and given the safety profile observed with other CatC inhibitors [28, 30], assessment of safety will include the occurrence of hyperkeratosis and periodontal disease as AESIs. A list of potential adverse events based on phase 1 trials of BI 1291583 can be found in the supplementary material.

Pharmacokinetic analysis of BI 1291583 will be performed using blood samples.

### Key inclusion and exclusion criteria

This trial will enrol patients with a computed tomography-confirmed diagnosis of bronchiectasis and a history of pulmonary exacerbations requiring antibiotic treatment. Patients with idiopathic bronchiectasis, as well as those with a broad range of underlying aetiologies, including, but not limited to, COPD, asthma, post-infectious rheumatoid arthritis or primary ciliary dyskinesia, will be eligible for this trial. In the 12 months before Visit 1, patients must have had either at least two exacerbations, or one exacerbation and be symptomatic as indicated by a SGRQ symptoms score of >40. The inclusion of symptoms in addition to exacerbation history is based on the observation that the risk of exacerbation increases by 10% for each 10-point increase in the SGRQ symptoms score as well as the demonstrated association between neutrophilic inflammation and symptoms in bronchiectasis [4, 31, 32]. Patients with cystic fibrosis and those with underlying diseases and/or treatments that represent an increased risk (*e.g.* those with a compromised immune system) are excluded from this trial. Patients with Papillon–Lefèvre syndrome (PLS) (a rare genetic disease with loss-of-function mutations in both alleles of the CatC gene), or hyperkeratosis of any aetiology, are likewise excluded from our trial, as are patients with medical conditions associated with periodontal disease (to be evaluated by a periodontist or dentist). Key inclusion and exclusion criteria are detailed in table 2. The full list is available in the supplementary material.

**TABLE 2:** Key inclusion and exclusion criteria

**Inclusion criteria**	**Exclusion criteria**
Male or female, aged ≥18 years^#^ and ≤85 years at screening	AST and/or ALT >3× ULN at Visit 1
Signed and dated written informed consent prior to admission to the study, in accordance with Good Clinical Practice and local legislation	Estimated glomerular filtration rate according to CKD-EPI formula <30 mL·min^−1^ at Visit 1
Women of childbearing potential^¶^ adhering to contraception requirements	Absolute blood neutrophil count <1000/mm^3^ at Visit 1
Clinical history consistent with bronchiectasis (cough, chronic sputum production and/or recurrent respiratory infections) and investigator-confirmed diagnosis of bronchiectasis by CT scan	Acute SARS-CoV-2 infection
History of pulmonary exacerbations requiring antibiotic treatment. In the 12 months before Visit 1, patients must have had either: 1) at least two exacerbations, or 2) one exacerbation and an SGRQ symptoms score of >40 at Screening Visit 1	Current smokers, or stopped smoking only within 3 months of screening, or not willing to maintain non-smoking status for the duration of the study
Current sputum producers with a history of chronic expectoration who are able to provide a spontaneous sputum sample at Screening Visit 1	Current diagnosis of cystic fibrosis, hypogammaglobulinaemia, common variable immunodeficiency, α1-antitrypsin deficiency, or allergic bronchopulmonary aspergillosis requiring treatment
	Any acute infections (including respiratory infections)
	Any mycobacterial infections, including pulmonary non-tuberculous mycobacterial disease, currently being treated
	Severe periodontal disease (to be evaluated by a periodontist or dentist) or palmar keratosis
	Any recent change in dose or regimen of maintenance bronchiectasis treatment
	Any medical condition that could interfere with participation or conduct of the study

AST: aspartate aminotransferase; ALT: alanine aminotransferase; ULN: upper limit of normal; CKD-EPI: Chronic Kidney Disease Epidemiology Collaboration; CT: computed tomography; SARS-CoV-2: severe acute respiratory syndrome coronavirus 2; SGRQ: St George's Respiratory Questionnaire. ^#^: ≥ 19 years in Republic of Korea; ^¶^: if allowed according to regulatory requirements.

### Recruitment and sample size calculation

With the planned sample size of 240 patients in a 2:1:1:2 allocation ratio, under the assumption of a hazard ratio of 0.5 for BI 1291583 5 mg once daily *versus* placebo, 0.6 for 2.5 mg once daily *versus* placebo and 0.8 for 1 mg once daily *versus* placebo, an overall power of at least 90% is achieved to demonstrate a non-flat dose–response curve in the dose-finding analysis and for the comparison of BI 1291583 5 mg *versus* placebo on the primary end-point. Overall exacerbation rates will be monitored blinded in order to potentially modify sample size and study duration (up to a maximum of 48 weeks), if needed. Such reassessment is important given the potential reduction in exacerbations observed during the COVID-19 pandemic and uncertainty over how long ongoing changes in behaviour will affect exacerbation rates [33].

### Planned analyses and assessments

The primary efficacy analysis consists of a multiple comparison and modelling (MCPMod)-based testing (with respect to a non-flat dose–response curve). MCPMod is used to evaluate several possible dose–response models (patterns), and to identify the best-fitting model or subset of models while keeping full control of the type I error at 0.05, one-sided. For the confirmatory testing of the primary end-point, the equality of the hazard ratios will be tested by the Wald test for the treatment effect in a Cox proportional hazards model at the one-sided 2.5% significance level. The effect of BI 1291583 5 mg *versus* placebo regarding the rate of pulmonary exacerbations will be analysed using a negative binomial model. In both models, the stratification factors will be included as covariates. Subgroup analyses will be carried out on the primary end-point according to baseline history of pulmonary exacerbations, baseline maintenance use of macrolides and *Pseudomonas aeruginosa* colonisation status. Details on the handling of missing data can be found in the supplementary material.

### Selection of doses

The human therapeutic dose is predicted from a minimum effective plasma area under the curve from 0 to 24 h (AUC_0–24_) of ∼340 nM·h in the preclinical mouse model [34]. Potency of BI 1291583 is threefold higher in humans compared with the mouse model, translating into an anticipated effective human plasma AUC_0–24_ of ∼110 nM·h. At repeat-dosing steady state, a dose leading to an AUC_0–24_ of 110 nM·h in humans is expected to result in 99% inhibition of CatC, and an AUC_0–24_ of 11 nM·h in 50% inhibition. An AUC_0–24_ of 110 nM·h is expected to be reached by 5 mg once daily and an AUC_0–24_ of 11 nM·h at 1–2.5 mg once daily, covering maximal and submaximal target engagement [35]. These predictions were confirmed by the phase 1 multiple-rising-dose study, and dosing was informed by BI 1291583 concentrations and NE inhibition in peripheral blood in single-rising-dose and multiple-rising-dose studies [27].

### Ethical approval

The study will be carried out in compliance with the protocol, the Declaration of Helsinki, the ICH Harmonized Guideline for Good Clinical Practice, relevant Boehringer Ingelheim Standard Operating Procedures, and other country-specific and relevant regulations.

## Discussion

Following promising preclinical [36] and phase 1 results [27], this phase 2 study of the novel CatC inhibitor BI 1291583 *versus* placebo in adult patients with bronchiectasis will determine the efficacy, safety and optimal dosing of BI 1291583 in this population. The study is designed to: 1) evaluate whether time to first exacerbation is prolonged and frequency of exacerbations is reduced by BI 1291583; and 2) evaluate whether clinical efficacy is associated with changes in NE activity in sputum.

BI 1291583 dose selection for this study was assisted by predictions from the preclinical mouse model of the human therapeutic dose expected to reach a required steady-state AUC_0–24_ of 110 nM·h, resulting in 99% inhibition of CatC. Calculations indicated that 5 mg once daily would achieve 110 nM·h, and 1–2.5 mg once daily would achieve 11 nM·h, resulting in 50% inhibition of CatC, covering maximal and submaximal target engagement [34]. Data from the phase 1 multiple-rising-dose study in healthy volunteers demonstrated that a 5 mg once daily dose of BI 1291583 exceeded predictions from preclinical studies, achieving a steady-state AUC_0–24_ of 187 nM·h at Day 28, and 1 mg and 2.5 mg once daily doses achieved steady-state AUC_0–24_ of 21.3 nM·h and 96.8 nM·h at Day 28, respectively [27]. Levels of peripheral blood neutrophil CatC inhibition attained at Day 28 for 5 mg once daily, 2.5 mg once daily and 1 mg once daily doses of BI 1291583 were 81.5%, 75.8% and 63.7%, respectively, and levels of peripheral blood neutrophil NE inhibition attained at Day 28 were 78.0%, 47.4% and 22.1%, respectively [27]. Differences in the achieved level of CatC inhibition compared with the predicted level (81.5% *versus* 99%) may reflect differences in neutrophil stimulation methodologies (lipopolysaccharide challenge in the mouse model *versus* zymosan stimulation in phase 1), harvesting of neutrophils (bronchoalveolar lavage in mice, peripheral blood in phase 1) and/or inter-species inflammatory responses.

Drug-related skin exfoliation was not reported more frequently in the BI 1291583 group than in the placebo group in the phase 1 trials [27]. This is an AESI that must be carefully monitored in clinical trials of CatC inhibition, as patients living with PLS exhibit palmoplantar hyperkeratosis and severe periodontitis [37]. It should be noted, however, that an equivalent 100% inhibition of CatC by a pharmacological agent and the highly reduced levels of CatC activity seen in patients with PLS are not expected. Hyperkeratosis was reported during phase 1 development of brensocatib (AZD7986, INS1007) [30], the only other CatC inhibitor in development for adults with bronchiectasis at the time of planning this phase 2 study (currently being investigated in the phase 3 study ASPEN (NCT04594369) [38]), and was related in a dose-dependent manner to the study drug. GSK2793660, an irreversible, competitive and selective CatC inhibitor for patients with bronchiectasis, exhibited similar potency to BI 1291583 at the preclinical stage [29, 34]. However, due to drug-related skin exfoliation events and modest pharmacodynamic efficacy, the development of this molecule was terminated at phase 1 [29]. In contrast to the early-phase findings of skin events with GSK2793660 and brensocatib, BI 1291583 did not increase risk of exfoliation in healthy volunteers participating in phase 1 studies [27]. This is despite a higher level of CatC inhibition *in vitro* with BI 1291583 than the active ingredient of brensocatib (INS1007), and in a mouse model up to 99% inhibition of the production of active NE (ED_50_ of 0.03 mg·kg^−1^) with BI 1291583 and an up to 76% inhibition (ED_50_ of 1.4 mg·kg^−1^) with INS1007 [36]. In the same model, we demonstrate that BI 1291583 preferentially distributes to the bone marrow by a maximum factor of 100 times compared with plasma, and INS1007 distributes almost equally between bone marrow and plasma [36].

The preferential distribution of BI 1291583 to the bone marrow may minimise the risk for skin events and is not expected to correlate with lower clinical efficacy. Indeed, a role for CatC in maintaining the structural integrity of plantar and palmar epidermal surfaces through processing of keratins in keratinocytes has been suggested [39]. This suggestion is supported by the observation of dose-dependent skin events in the phase 1 study of brensocatib [30] – the relative rapidity in the onset of these events did not correlate with the dynamics of NE activity. Further, in the terminated phase 1 study of the irreversible CatC inhibitor GSK2793660 [29], marked skin desquamation events were observed in the absence of inhibition of NSP activation.

In the phase 1 study of brensocatib [30], levels of blood NE activity inhibition achieved after 28 days at 25 mg once daily, the highest dose used in WILLOW – a 24-week phase 2 study of 10 mg once daily and 25 mg once daily brensocatib in patients with bronchiectasis [28] – were ∼50%. In WILLOW, decreases in sputum NE activity, reductions in risk of exacerbation over the treatment period and decreases in annualised exacerbation rate, compared with placebo, were observed [28]. As BI 1291593 5 mg once daily achieved a mean maximal 78% inhibition of NE activity in blood [27], high NE activity inhibition in sputum resulting in clinically relevant benefits is also expected to be achieved.

The 48-week duration of Airleaf™ is a key strength. The incidence of skin- and dental-related adverse events in WILLOW [28] was higher in the brensocatib groups than in the placebo group, although it should be noted that the incidence of the AESIs hyperkeratosis and periodontitis was comparable across groups. Furthermore, no evidence of higher rates of infection was observed. However, conclusions about the longer-term efficacy and safety of brensocatib are difficult to make due to the 24-week duration of the WILLOW study. The 48-week duration of our study improves the robustness of any conclusions about the efficacy and safety of BI 1291583. The variable 24- to 48-week treatment period also allows all patients, once randomised, to be treated for at least 24 weeks and up to 48 weeks.

A further key strength of our study is the inclusion of patients with a history of only one exacerbation requiring antibiotic treatment and an SGRQ symptoms score of >40. The inclusion of the symptoms score enriches for future exacerbations in any patients emerging from COVID-19 lockdown where a potential reduction in observed exacerbations may occur, as the risk of exacerbation is not predicted just by history of exacerbations but also by symptoms [31]. In addition, the sample size adjustment allowed by our study is especially important because associated social isolation features may result in potential future reductions in observed exacerbations [33].

The key differentiating factor in our study is the inclusion of a novel broad patient population with regard to underlying aetiologies in Airleaf™, specifically the inclusion of patients with a primary diagnosis of asthma and COPD. Such patients have generally been excluded from bronchiectasis studies. They are included here as active neutrophilic inflammation is the driver of bronchiectasis in these patients and therefore targetable for medication, and their inclusion allows us to investigate a precision medicine approach targeting patients with bronchiectasis in need of an anti-inflammatory treatment. This recognises that these are patient populations with a high burden of illness and neutrophilic inflammation that may benefit from a CatC inhibitor [40–42]. In addition, bronchiectasis as a primary diagnosis may be missed in patients who smoke and are, therefore, labelled with COPD [40, 41]. In either case, the underlying neutrophilic inflammatory processes may benefit from CatC inhibition.

### Conclusion

This study aims to evaluate the efficacy, safety and optimal dosing of the novel CatC inhibitor BI 1291583 in adults with bronchiectasis. If efficacy and safety are demonstrated, results will support further investigation of BI 1291583 in phase 3 trials.

## Supplementary material

10.1183/23120541.00633-2022.Supp1 **Please note:** supplementary material is not edited by the Editorial Office, and is uploaded as it has been supplied by the author.
Supplementary material 00633-2022.SUPPLEMENT
